# Achieving Family-Integrated Care for Older Patients with Major Neurodegenerative and Mental Health Conditions: A Systematic Review of Intervention Characteristics and Outcomes

**DOI:** 10.3390/ijerph22071096

**Published:** 2025-07-10

**Authors:** Shruti Jindal, Mohammad Hamiduzzaman, Harry Gaffney, Noore Siddiquee, Helen McLaren

**Affiliations:** 1 Department of Psychology, Jamia Millia Islamia University, Jamia Nagar, New Delhi 110025, India; 2 Faculty of Medicine and Health, The University of Sydney, Sydney, NSW 2006, Australia; 3 Faculty of Arts, University of Melbourne, Melbourne, VIC 3010, Australia; 4 School of Allied Health, Australian Catholic University, Melbourne, VIC 3065, Australia

**Keywords:** family integration, models of care, older patients, neurodegenerative conditions, mental health

## Abstract

National and international aged care frameworks recommend family-integrated care to enhance care quality and outcomes, supported by evidence demonstrating improvements in patient and clinician experiences. Yet uncertainty remains about how to integrate family carers effectively in diverse healthcare models and settings for neurodegenerative and mental health conditions. A systematic integrative review was conducted to answer two research questions: how do the studies describe the integration of family carers in health services design and delivery for older patients with neurodegenerative and mental health conditions? And what is the evidence for family-integrated care models impacting the health and wellbeing of these older patients? Structured and iterative searches of five databases (CINAHL, Medline (Ovid), Web of Science, PsycINFO, and ProQuest) and the Google Scholar search engine identified 2271 records. A Covidence screening process resulted in 14 studies for review, comprising randomised controlled trials, mixed methods studies, qualitative studies, and quasi-experimental designs. The following four themes emerged from the evidence synthesis: (1) family participation in service delivery, (2) health and wellbeing outcomes, (3) satisfaction with care, and (4) service dynamics in enabling family-integrated care successfully. This review highlights that while family-integrated care models contribute to positive health and wellbeing outcomes for older patients with neurodegenerative and mental health conditions, challenges remain for implementation due to the extent and variability in integration strategies, a lack of rigorous evaluation, and an absence of standardised frameworks.

## 1. Introduction

Globally, an estimated 20–30% of older adults (≥65 years) are affected by neurodegenerative conditions [1], while approximately 14% are diagnosed with a mental health disorder. This review focuses on the major neurodegenerative conditions, such as Alzheimer’s disease, dementia, and Parkinson’s disease, as well as common mental health conditions, including depression and anxiety [2]. These conditions are selected due to their high prevalence among older populations and their association with poor health outcomes [1]. Both neurodegenerative and mental health conditions significantly impair functional independence and quality of life [3]. Importantly, these conditions frequently co-occur and co-exist; for instance, it is estimated that 60–80% of older adults with dementia also experience depression and/or anxiety [4]. Such co-occurrence of neurodegenerative and mental health conditions is linked to adverse health and social outcomes, including increased risk of falls, reduced mobility, accelerated cognitive decline, social isolation, and hospitalisation [5]. This complex interplay increases the demand for long-term, coordinated healthcare and support services to manage physical and psychological needs [5].

Providing timely and sustained care for older patients with neurodegenerative and mental health conditions (OPNMHCs) presents an ongoing challenge across acute and non-acute care settings. In most healthcare systems, primary care is central in delivering aged care services, typically through general practices, community health centres, aged care facilities, and in-home care programs. These services provide person-centred, continuous care that addresses both physical and mental health needs, promoting independence and quality of life. Acute care settings are often involved during periods of crisis, symptom exacerbation, or when diagnostic, specialist, or intensive interventions are required. Current healthcare models used in primary care and hospital settings increasingly emphasise not only the treatment of illness but also prevention strategies, comprehensive management, and rehabilitation [6]. Despite increasing prevalence and growing needs among OPNMHCs, many existing healthcare models lack the flexibility and integration to deliver consistent, holistic care for this cohort.

Integrating family carers into the design and delivery of healthcare services has emerged as a promising approach to enhance person-centred aged care. As defined by Carers Australia [7], family carers are “people who provide unpaid care and support to family members and friends who have a disability, mental illness, chronic condition, terminal illness, an alcohol or other drug issue or who are frail aged”. Family carer engagement in care planning and service delivery has been linked to improved continuity of care, greater responsiveness to patient needs, and better satisfaction with services [7,8]. Several programs are available that support and formalise family integration. These models vary in their approach; for example, the Family-Integrated Care (FICare) Model primarily focuses on empowering parents as primary caregivers in neonatal settings, but its principles are being adapted elsewhere [9]. In contrast, the Fam-FFC (Family-Focused Functional Care) Model specifically targets the engagement of family in promoting functional recovery in hospitalised older adults [9]. Other models are more educational or skills-based, such as the REACH VA (Resources for Enhancing Alzheimer’s Caregiver Health) and Savvy Caregiver programs, which provide training and support to improve the health and capabilities of dementia caregivers [10]. Family-integrated care (FICare) is a model of care that actively involves family members in the care planning, decision-making, and delivery process. This collaborative approach aims to enhance the quality of care by leveraging the expertise and support that family carers offer [11]. However, current family-integrated care models are often not tailored to the complex and overlapping needs of older patients with neurodegenerative and mental health conditions. The existing literature is limited in addressing the integration of family carers in care models that manage both physical and psychological health challenges, especially in the presence of co-occurring conditions like dementia and depression [12]. While these programs have demonstrated benefits in improving family carer knowledge, reducing stress, and enhancing functional outcomes for care recipients, most were not explicitly designed to address the complex and overlapping needs of OPNMHCs, particularly those with co-occurring neurodegenerative and mental health conditions. High-quality evaluation of family-integrated care remains limited due to small sample sizes, high attrition rates, variation in interventions, and inconsistent outcome measures. Additional barriers in the robust assessment include carer fatigue, time constraints, and the exclusion of older patients with acute health conditions, resulting in fragmented insights into the impact of family-integrated care for OPNMHCs.

Various international and national policies and frameworks, such as the WHO’s Global Strategy and Action Plan for Ageing and Health (2016–2020) [13], the Australian Government’s Aged Care Quality and Safety Commission Fact Sheet [14], and the Partnerships in Care Program [15]—promote collaboration with families in aged care. Still, they are not yet fully tailored to address the complex needs of OPNMHCs. There are scoping and systematic reviews of family integration policies, frameworks, and programs in the care of older patients in acute and non-acute healthcare settings [16,17,18,19,20,21]. Limited reviews report a small body of evidence explaining the characteristics and outcomes of family-integrated care for OPNMHCs. To ensure that family carers are meaningfully integrated in care design and delivery for OPNMHCs, synthesising current evidence on family integration practices is important. A targeted review of family-integrated care models in hospital and community-based contexts would help clarify the characteristics of available evidence, highlight consistent and divergent findings across primary studies, and examine reported health and wellbeing outcomes, as well as the alignment of family carer integration strategies across different models of care.

Our review aimed to identify evidence of the impact of family-integrated care models on community-dwelling and hospital-admitted OPNMHCs, as well as to determine which family integration methods best meet patients’ complex care needs in different settings. The PICO framework was employed to formulate the review questions [22].

P (Population)—older patients (≥65 years) with neurodegenerative and mental health conditions (OPNMHCs);

I (Intervention)—family-integrated care models;

C (Comparison)—not applicable;

O (Outcomes)—service dynamics, meaning family integration in the health services delivery, and effectiveness, meaning health outcomes and satisfaction with care.

Two main questions guided this review, as follows:

- How do the studies describe the integration of family carers in health services delivery for community-dwelling and hospital-admitted OPNMHCs?
- What is the evidence of impact that family-integrated care models produce on OPMHNCs’ health and wellbeing?

## 2. Materials and Methods

### 2.1. Review Design

The process followed in this review involved identifying the issue, conducting a search for relevant literature, critically evaluating the data, synthesising the findings, and presenting the results. Cooper initially outlined this integrative review approach [23], later updated by Whittemore and Knafl [24]. To capture the full breadth of evidence on this complex, multifaceted topic, we included a wide variety of study designs, including randomised controlled trials, quasi-experimental studies, qualitative studies, and mixed methods studies. This approach is central to the integrative review methodology, which aims to synthesise diverse forms of evidence to create a more holistic understanding of a phenomenon. A systematic literature search was conducted, and the Mixed Methods Appraisal Tool (MMAT) was applied to evaluate the quality and robustness of the selected studies [25]. The extracted data were then synthesised and presented thematically.

### 2.2. Search Strategy

A systematic search for published literature was conducted in accordance with the PRISMA guidelines [26] (Supplementary File S1: PRISMA Checklist). Two authors (SJ and MH) independently conducted searches across five electronic databases—CINAHL, Medline (Ovid), Web of Science, PsycINFO, and ProQuest—as well as the Google Scholar search engine—in April 2024 and updated the search in February 2025. Searching concepts, keywords, and MeSH headings were developed based on evidence scoping and reviewed with a university librarian. The major concepts used in the search were “older patients”, “neurodegenerative and mental health conditions”, “family-integrated care”, and “community and hospital settings” (the full search strategy is available in Table 1, adapted to the requirements of respective databases and search engines).

### 2.3. Inclusion and Exclusion Criteria

The inclusion and exclusion criteria are presented in Table 2. We aimed to select empirical studies that used randomised controlled trials (RCTs), pre–post-test, longitudinal, quasi-experimental, cross-sectional, and case-control research designs, which investigated OPNMHCS, their family carers, and healthcare professionals’ experiences and perspectives on family-integrated care models between 2005 and 2025. We focused on neurodegenerative and mental health conditions, such as Alzheimer’s, dementia, Parkinson’s disease, depression, and anxiety in the study selection. Studies involving conditions such as Huntington’s disease and acute, non-progressive neurological illnesses were excluded, because these conditions differ significantly in their clinical management and trajectory compared to neurodegenerative and mental health conditions, which are the primary focus of this review. To ensure a consistent and clear selection criterion, we operationalised the definition of family-integrated care based on the core components identified in the literature [27]. For a study to be included, the intervention had to involve family carers in at least one of the following three capacities as part of a formalised care model or program: (1) active participation in care planning, goal setting, or shared decision making; (2) delivery of direct physical, emotional, or therapeutic support that was guided or structured by healthcare professionals; or (3) formal involvement in care coordination activities. This operational definition guided our decisions on inclusion and exclusion.

The search results were exported to EndNote, and duplicates were removed. The reference list was then imported into Covidence. A two-phase screening process was undertaken. In the first phase, two reviewers (SJ and MH) independently screened the titles and abstracts of all identified records against the inclusion criteria. In the second phase, the same two reviewers independently assessed the full text of the potentially eligible studies to determine final inclusion. Any disagreements at either phase of the screening process were resolved through discussion and consensus between the two reviewers (SJ and MH). A third reviewer (HG) was available to arbitrate any unresolved disagreements, though this was not required. Although a formal kappa statistic was not calculated, a high level of initial agreement was observed, and the consensus process ensured that all inclusion decisions were rigorously vetted and agreed upon.

### 2.4. Quality Appraisal

The quality of the included studies was evaluated using the 2018 version of the Mixed Methods Appraisal Tool (MMAT) [25]. The MMAT was specifically chosen because it is one of the few quality appraisal tools designed to concurrently assess the methodological quality of a wide range of study designs, including qualitative, quantitative (randomised and non-randomised), and mixed methods studies, within a single instrument. Given the intentional inclusion of diverse study types in this integrative review, using design-specific tools such as the Cochrane Risk of Bias tool (for RCTs) or the Newcastle–Ottawa Scale (for non-randomised studies) would have been disjointed and prevented a consistent appraisal approach across the evidence base. The MMAT provides a coherent framework for this purpose. The tool scores studies from 0 (indicating that no criteria are met) to 5 (indicating that all requirements are met), following the methodology outlined by Hong et al. [25]. While tools recommended by Cochrane are the gold standard for their respective study designs (e.g., RoB 2 for RCTs), the MMAT is recognised as a valid and reliable tool for the specific context of reviews that synthesise mixed study designs [25], which was the explicit purpose of this review.

The quality of the included studies was assessed independently by two reviewers (SJ and MH). Any disagreements between the reviewers regarding study quality were resolved through discussion. A final consensus was reached, ensuring that the reliability of the quality appraisal process was maximised. The first reviewer, SJ, assessed the studies, assigning scores ranging from 0 (Unclear/No) to 1 (Yes). Through consensus, it was determined that the studies included in this review ranged in quality from low to high, as detailed in Table 3.

### 2.5. Data Extraction and Analysis

Two reviewers, SJ and MH, conducted multiple readings of the studies that met the inclusion criteria to facilitate data extraction. The reviewers used separate Excel sheets to record their findings, carefully cross-checking discrepancies (see Table 3). Extracted data included study characteristics such as author, year, country, study design, and participant details, as well as specifics related to family-integrated care for OPNMHCs (e.g., focus of care models, mechanisms for family integration, and level of family involvement in service delivery). Outcome data related to OPNMHCs, including demographics, health outcomes, and satisfaction with care, were also extracted. A reflexive thematic synthesis accommodates the varied methodologies in the included studies [28]. For qualitative studies, data extraction included thematic coding of family carer involvement, the nature of interventions, and patient outcomes. A reflexive thematic synthesis, as described by Braun and Clarke [28], was conducted to analyse and synthesise the findings from the included studies. This approach was chosen for its flexibility in accommodating the varied methodologies in the included evidence [28]. The analysis was performed by two reviewers (SJ and MH) and followed a rigorous, multi-step process. First, both reviewers independently familiarised themselves with the full text of all 14 included studies to gain an in-depth understanding of the data. Second, the reviewers independently generated initial codes from the data, focusing on aspects related to family integration, service dynamics, and outcomes. Third, the reviewers met to discuss, compare, and collate their initial codes into potential themes. Through a series of discussions, these themes were reviewed, refined, and organised into a coherent thematic framework. Regular team meetings with the wider research team (HG, NS, HM) were held to discuss and challenge the developing themes, ensuring the final interpretations were robust, well-grounded in the data, and consistently categorised. The final four themes presented in this review were confirmed through this collaborative and iterative process.

**Table 3:** Studies evaluating the impact of family-integrated models of care for older patients with neurodegenerative and mental health conditions.

Author (Year) and Country	Study Design	Study Settings	Study Participants	Integration of Family in Service Delivery			Outcomes			MMAT Scores
				Focus on Family-Integrated Care	Mechanisms into Family Integration	Level of Integration in Service Provision	Demographics and Health Problems Dealt with	Health and Wellbeing Outcomes	Satisfaction with Care Involving Family Carers	
Fortinsky et al., (2020); USA [29]	Stratified randomization design; psychometric testing, telephonic interviews	Community settings, including in-home care for community-dwelling older adults	576 members (participants included older adults aged over 65 with cognitive vulnerability, such as dementia, depression, and/or delirium) and 380 caregivers	To help shape the care model, provide personal experience and expertise, and refine the intervention	- Patient-oriented research approach - Family members participated in an advisory group - Co-designing with collaborative efforts between researchers, family members, and healthcare providers	Screening and Clinical Assessment: - Using tools like the Family Confusion Assessment Method (FAM-CAM) for delirium screening and the AD-8 for dementia screening - Use of ICD-10 diagnostic codes and clinical assessment tools, such as the Telephone Interview for Cognitive Status (TICS) Treatment Plan Development: - Families shared their preferences, residents’ preferences, and identifying residents’ problems - Family input in medication review and reconciliation, physical therapy inclusion, and overall care management strategies Treatment Plan Implementation: - Continuous involvement of family members in the care process, communication with the 3D Team, and participation in interventions, such as Problem-Solving Therapy and home-based physical therapy - Coordination of services and follow-ups facilitated by family caregivers and healthcare providers	Demographics: - Target population: adults over 65 years old - Insurance: insured by a Medicare Advantage (MA) plan - Living situation: members living at home - Health conditions: dementia, depression, recent episode of delirium - Reference term: referred to as members in the trial - Total enrolment: 576 members will participate - Intervention groups: 3D Team Intervention group, Usual Care group (MA plan’s existing care management program) Health Problems Dealt With: Cognitive vulnerability, dementia, depression, delirium	Statistically Significant Outcomes: A prior intervention that included home assessments and visits for older adults at high risk of hospitalization showed a 7–38% reduction in similar outcomes over a 1–2-year period Psychological Wellbeing Outcomes: - Positive outcomes included improvements in patient functioning, prevention or delay of behavioural problems (e.g., agitation), caregiver wellbeing, reduction in caregiver burden, caregiver knowledge of dementia care management strategies - Collaborative care models: Utilized Problem-Solving Therapy and other interventions; showed promising effects in reducing depressive symptoms, slowing down functional disability declines, reducing suicidal ideation	Involves primary care providers and mental health specialists, Problem-Solving Therapy Successful Outcomes: Reduction in depressive symptoms and other health-related outcomes important to patients Patient Satisfaction: Study participants reported high levels of satisfaction with treatment provided by clinicians trained as depression care specialists	5
Boltz et al., (2014); USA [30]	Comparative repeated measures design; psychometric testing (pen and paper questionnaire)	Three medical units of a community teaching hospital in the Northeast US	97 dyads (patients and FCGs)	- Family caregivers (FCGs) can exert considerable influence over the older adult’s care delivery and activity level - Older adults are more likely to engage in self-care and walking programs when encouraged and advocated for by family members - Hospitalization often causes strain, anxiety, depression, and stress in FCGs, affecting their relationship with the care receiver - Interventions that support the role of FCGs in the functional recovery of hospitalized older adults warrant attention	- Fam-FFC intervention - Systematic inclusion of FCG and patient in the assessment and jointly developed bedside goals and care plans	Screening: - FCGs were involved in care through information sharing (96%), decision making (94%), companionship (92%), advocating (72%), coordination (64%), and direct care (56%) - Systematic inclusion in assessment, including baseline cognition and physical function Treatment plan development: Jointly developed bedside goals and care plans that support functional recovery and prevention of complications, updated daily with assigned nursing staff, FCG, and patient Treatment plan implementation: FCGs were involved in direct care activities, including cognitive stimulation, assistance with meals, bathing/showering, hygiene, and walking Rehabilitation: Emphasis on collaboration with other disciplines, including rehabilitation Tools used in service delivery: Discharge teaching and planning incorporating function-focused care dimensions in addition to management of the medical condition	Patient Characteristics: - The majority of patients were female (53%) - Most patients were white (89%) - Marital status: married (51%), widowed (40%) - Mean age: 80.8 (± 7.5) years - Education: 33% of patients were college educated - Living situation: 95% were admitted from a private residence; the rest were admitted from assisted living - Barthel Index (self-reported, 2 weeks prior to admission): 91.1 (±17.0) - Mobility: 43% of patients used an assistive device for mobility Family Caregiver (FCG) Characteristics: - The majority of FCGs were daughters (34%), followed by female spouses/partners (31%) - Marital status: 78% of FCGs were married - Education: 42% had college education or higher - Age range: 38% of FCGs were aged 46–65 years - Employment: 54% of FCGs were employed Health Problems Dealt With: Cognitive impairment: delirium, depression	Statistically Significant Outcomes: - Delirium severity: repeated measures ANOVA demonstrated that the intervention was associated with a significant decrease in overall delirium severity (F(1.3) = 3.5, *p* = 0.05); post hoc tests revealed that the Fam-FFC arm showed less delirium severity from admission to all other time points - ADL (activities of daily living) performance: Patients in the Fam-FFC group demonstrated better ADL performance (F(2.3) = 3.3, *p* = 0.03), with improvement evident at 2 months after discharge - Walking performance: mean walking performance differed significantly between intervention arms (F(2.5) = 3.6, *p* = 0.02); post hoc tests revealed less decrease in walking performance at 2 months post-discharge for the Fam-FFC group Non-Statistically Significant Outcomes: There was no significant effect of the intervention on Tinetti Gait and Balance (F(1.4) = 1.9, *p* = 0.173) Psychological Wellbeing Outcomes: - Anxiety: mean FCG anxiety differed significantly between groups (F(1.9) = 9.4, *p* < 0.0001); the Fam-FFC intervention was associated with less anxiety from admission to two months post-discharge; in the non-intervention group, the percentage of FCGs experiencing anxiety increased from 16% at admission to 34% at discharge (*p* = 0.011); in the intervention group, anxiety decreased from 36% at admission to 22% at discharge (*p* < 0.0001) - Depression: mean depression scores differed significantly between intervention arms (F(2.1) = 4.4, *p* = 0.012); in the Fam-FFC group, depression decreased from 22% at admission/discharge to 10% two months post-discharge (*p* < 0.0001); in the non-intervention group, depression increased from 11% at admission to 13% two months post-discharge (*p* = 0.023) Physical Wellbeing Outcomes: Physical function improvements in the intervention group were significant, with better ADL performance and walking performance but no significant improvement in gait/balance compared to the control group	- The intervention was associated with a significant increase in preparedness for caregiving (F(2.6) = 4.4, *p* = 0 007) - Post hoc tests revealed that the Fam-FFC group was associated with increased preparation for caregiving from admission to two months post-discharge	2
Dening et al., (2019); UK [31]	User-centred design (UCD) methodology (5)	Dementia UK and De Montfort University in Leicester, UK	Stakeholder group sizes: - Family carers (n = 8) - People with dementia (n = 3) - Admiral nurses (n = 11) - Palliative care consultant (n = 1) - Specialist palliative care nurse (n = 1) - Other contributors (e.g., facilitators, professors, assessors)	To ensure that care planning reflects the practical and emotional needs of both patients and caregivers, aiming to reduce stress and improve outcomes	A user-centred design (UCD) methodology was employed to develop the ACP guide and template. This method involves active involvement of end users (families affected by dementia) during the analysis and design process Focus groups, electronic feedback, and iterative testing with families were part of the process to refine the ACP guide and template	- The family was integrated because of their critical role in providing day-to-day care for people with dementia. Including families ensures that care planning reflects the practical and emotional needs of both patients and caregivers, aiming to reduce stress and improve outcomes Tools Used for Screening: The study used a co-design approach under the UCD methodology, actively involving families in every step of creating the ACP tools Feedback was gathered iteratively from families, professionals, and stakeholders Screening/Clinical Assessment: focusing on identifying preferences and needs through structured discussions, but it did not involve clinical diagnostic tools. Treatment Plan Development: - Families were actively involved in co-designing the treatment plan by sharing their preferences and experiences to guide the development of the ACP tools Treatment Plan Implementation: Families participated in implementing the treatment plan by field-testing the ACP guide and providing feedback on its usability and relevance Tools used: Tools like structured communication protocols and the ACP guide/template were used to support integration	Demographics: - The study involved people with dementia who are members of Dementia UK’s Patient and Public Involvement Group (supported by the group facilitator) (n = 3), family carers (n = 8), and professionals, including admiral nurses (n = 11), a palliative care consultant (n = 1), and a specialist palliative care nurse (n = 1) Health problems dealt with: - Dementia, focusing on its cognitive, communicative, and functional decline. Secondary focus areas included anxiety and planning for anticipated health deterioration Physical Wellbeing Outcomes: Indirect physical wellbeing outcomes were implied through improved caregiving coordination and communication	Statistically Significant Outcomes Positive health outcomes include improved communication, relationship maintenance, and reduced psychological stress. These contribute to the quality of life but are not described with statistical significance Psychological Wellbeing Outcomes: reduced anxiety and enhanced coping abilities for families and patients. ACP discussions help mitigate stress and improve emotional preparedness	- Satisfaction with care - Feedback from families indicated satisfaction with the usability and feasibility of the ACP guide and template, particularly noting that it reflected their input and preferences	5
Gitlin et al. (2008); USA [32]	A prospective, two-group controlled pilot study	Home settings	60 dyads (dementia patients and their caregivers)	- To reduce behavioural disturbances in dementia patients and caregiver burden - To enhance patient engagement, caregiver mastery, self-efficacy, and use of simplification strategies To address caregivers’ concerns about occupying their relatives and supporting self-identity	- Tailored Activity Program (TAP) involved 8 sessions, six home visits (90 min each) and two (15 min) telephone contacts by occupational therapists (OT) over 4 months. Contacts were spaced to provide caregivers opportunities to practice using activities independently - In the first two home sessions, interventionists met with caregivers, introduced intervention goals, used a semi-structured investigator-developed interview to discern daily routines, and the Pleasant Event Schedule to identify previous and current activity interests. Tools used: interventionists observed dyadic communication and home environmental features and assessed dementia patients using the Dementia Rating Scale and Allen’s observational craft-based assessments	- Screening: Participants were recruited through media announcements and mailings by social service agencies. Interested caregivers contacted the research office, were explained study procedures, and administered a brief telephone eligibility screen - Treatment Plan Development: In subsequent sessions, interventionists identified three activities and developed 2–3-page written plans (Activity Prescriptions) for each. Each prescription specified patient capabilities, an activity and goal, and specific implementation techniques. Caregivers, and when appropriate dementia patients, chose one activity prescription to focus on first. The prescription was reviewed, and the activity introduced through role-play or direct demonstration with patients - Treatment Plan Implementation: Caregivers were instructed in stress-reducing techniques to help establish a calm emotional tone. Caregivers practiced using the activity between visits. Once an activity was mastered, another was introduced. In each session, prescriptions were reviewed and modified if necessary - Rehabilitation: - Enhancing caregiver skills and reducing their objective burden through activity use	Demographics: - The study involved 60 dyads (dementia patients and their caregivers) - Dementia patients: primarily male (57%), white (77%), mean age of 79 years - Caregivers: primarily female (88%), white (77%), high school graduates (56%), spouses (62%), mean age of 65 years Health Issues Dealt With: dementia	Statistically Significant Health Outcomes: - Behavioural occurrences: a treatment effect was found for frequency of behavioural occurrences (*p* = 0.009; Cohen’s d = 0.72); reductions in shadowing: adjusted mean difference = −1.00, CI = −1.36 to −0.64 (*p* = 0.003, Cohen’s d = 3.10); reductions in repetitive questioning: adjusted mean difference = −0.49, CI = −0.90 to −0.07 (*p* = 0.023, Cohen’s d = 1.22) - Agitation and argumentative behaviours: statistically significant reductions in agitation (*p* = 0.014, Cohen’s d = 0.75) and argumentative behaviours (*p* = 0.010, Cohen’s d = 0.77) were observed compared to controls Non-Statistically Significant Health Outcomes: Depressed mood: there was no significant effect found for depressed mood; there was a slight decrease in the number of reported behaviours for TAP participants compared to controls (for whom the number of behaviours increased), but the difference did not reach statistical significance Psychological and Physical Wellbeing Outcomes: Tailored activities: activities tailored to the cognitive capacity and interests of dementia patients resulted in symptom reduction by enhancing role identity and helping dementia patients express themselves positively; life-long activities were modified to match patient abilities, minimizing frustration and fostering positive engagement	- 84.8% of caregivers indicated that the intervention was very useful, with 15.2% finding it somewhat useful; 89.1% of caregivers indicated that the intervention had a positive effect; only 10.9% indicated that strategies had no effect or made matters worse	2
Budgett et al., (2024); UK [33]	Randomized controlled trial (RCT) with qualitative content analysis	Urban and regional areas across England, including National Health Service (NHS) trust memory clinics, older adult mental health services, general practitioner practices	People Living with Dementia (PLWD): n = 302 Family carers: n = 302	To explore priorities related to living well at home: - Participants set goals around what would help the PLWD to live for as long and as well as possible in their own homes - The goal-setting process prioritized carers’ input when PLWD lacked capacity, aiming to identify key areas where families could directly support PLWD - Carers often reported unmet needs related to respite, worry, and coping strategies, which justified their inclusion as they are critical in supporting PLWD’s quality of life and care	- Goal Attainment Scaling (GAS): GAS was used collaboratively by nonclinical facilitators to set goals with family carers and PLWD - Patient-Oriented Research Approach: Facilitators were trained to engage both the carer and PLWD in open-ended yet focused discussions to set SMART goals tailored to individual needs - Co-Design of Services: - Carers were engaged in designing care goals relevant to both their own and the PLWD’s priorities	Screening as Part of Clinical Assessment: Baseline assessments involved identifying the unmet needs of PLWD and carers, including cognitive, mood, and behavioural domains - Treatment Plan Development: Goals were tailored based on GAS discussions that prioritized family input to co-create relevant, attainable care objectives - Treatment Plan Implementation: Carers implemented certain intervention aspects, such as supporting activities for mood improvement or assisting with personal care strategies - Rehabilitation: Rehabilitation goals were primarily indirect, focusing on managing behaviours or enhancing PLWD’s social interactions, supported by carers - Tools Used: SMART (Specific, Measurable, Attainable, Relevant, Time-bound) goals were formulated using structured GAS frameworks - Additional tools included fidelity checklists to ensure goals aligned with family priorities and feasibility assessments during follow-ups	Demographics: Participants: People Living with Dementia (PLWD): n = 302 Family carers: n = 302 Age: PLWD: mean age = 79.9 years Carers: mean age = 63.4 years Gender: PLWD: Male = 44%, Female = 56% Carers: Male = 29.8%, Female = 70.2% Ethnicity: Majority White (88.1%), with smaller representations from Mixed (1.3%), Asian (5.6%), and Black (3.6%) communities. Dementia Diagnoses: Alzheimer’s disease = 46.0% Vascular dementia = 12.6% Lewy body dementia = 3.3% Frontotemporal dementia = 2.6% Other = 27.8% Unspecified = 7.6% Health Problems Addressed: Mood: Reducing anxiety/worry and improving interest/initiative in activities for better mental stimulation Behaviour: Increasing positive interactions and reducing or coping with repetitive or unsafe behaviours Self-Care: Goals included managing pain, improving mobility, and addressing resistance or distress during personal care Cognition: Improving memory, managing repetitive questions, and coping with concentration or orientation problems Physical Health: Managing limited walking ability and promoting activities like leaving the house twice a week Improving fluid intake and appetite to address weight loss or other nutritional concerns	Statistically Significant Outcomes: Improvement in Mood: Goals in this domain focused on improving the PLWD’s interest, initiative, and engagement in activities to improve mood One goal aimed for a PLWD to ‘leave the house without anxiety symptoms lasting longer than 3 min, twice a week or more’ Positive Behaviour Changes: Increasing positive social interactions with others was a key goal, such as ‘having social contact with family or friends (other than the primary carer) once a week for at least 15 min’ Self-Care and Mobility: A goal for self-care included ‘reducing distress during personal care sessions’ and improving physical health, such as ‘getting out of the house at least two times a week’ Psychological Wellbeing Outcomes: Goals related to reducing anxiety, such as ‘PLWD can leave the house without anxiety symptoms lasting longer than 3 min,’ showed significant psychological benefits Goals also targeted reducing frequency or intensity of low mood and engaging PLWD in stimulating activities Physical and psychological outcomes were linked, e.g., improving mobility to participate in activities contributed to psychological wellbeing	Satisfaction with Interventions: - Facilitators noted that involving family carers in setting personalized goals enhanced satisfaction by aligning care interventions with the needs of both PLWD and carers - Caregiver satisfaction improved through involvement in co-designing and delivering interventions, such as ‘implementing supportive strategies for PLWD’s personal care or managing repetitive behaviours’ - Family members valued the structured process, which facilitated goal attainment and highlighted personalized care	5
Gitlin et al., (2001); USA [34]	Randomized, controlled trial	Home settings	171 family caregivers of dementia patients	To provide caregivers with practical skills and mechanisms to exert control over difficult situations, targeting caregiver upset and self-efficacy	- Educating caregivers about the impact of the environment on dementia-related behaviours and simplifying objects in the home to reduce agitation and improve daily functioning - Occupational therapists provided education about dementia, engaged caregivers in mutual problem solving, introduced environmental simplification and task breakdown strategies, and used cognitive restructuring to instil greater perceived control and confidence in caregivers	- Screening: Caregivers reported on the frequency of behavioural problems and the level of dependency in ADLs and IADLs of the person with dementia using standardized measures, such as the Memory and Behaviour Problems Checklist and the Functional Independence Measure (FIM) - Treatment Plan Development: The occupational therapists worked with caregivers to develop targeted plans addressing specific aspects of daily care, provided education about the disease process, and engaged in mutual problem solving with caregivers to identify alternate care strategies - Treatment Plan Implementation: Caregivers were coached to implement environmental strategies, observed using recommended strategies, and received refinements and new recommendations during home visits Tools: environmental simplification and task breakdown strategies were primary methods used during the intervention - Rehabilitation: The intervention aimed to slow the decline in functional independence (ADLs and IADLs) of dementia patients	Demographics: - Baseline characteristics of participants in both the experimental and control groups showed no large or significant differences at baseline - The sample was primarily female, married, and had a high school or higher education - Of the 171 participants, 126 (74%) identified as White, 43 (25%) identified as African American, 1 caregiver identified as Hispanic, 1 caregiver identified as Other - Spouse caregivers represented 25% of the sample; daughters and daughters-in-law constituted 59% of the sample; sons, sons-in-law, and grandsons represented 13% of the sample; other family relationships (e.g., nephew) made up 3% - Caregivers were on average 61 years old (range 23 to 92 years) - Caregivers reported providing care for an average of 45 months (range 2 months to 16 years) Health Problems Dealt With: - Alzheimer’s disease or a related disorder Dementia-related behaviours (e.g., wandering, agitation)	Statistically Significant Outcomes: Regarding outcomes related to dementia patients, there was a statistically significant effect in one of the three outcomes studied; caregivers in the experimental group reported less decline in IADL dependence in the person with dementia compared to the control group caregivers (*p* = 0.03) Non-Statistically Significant Outcomes: - There was a trend toward less decline from baseline to post-test for behaviours and ADL dependence, although these were not statistically significant - There were no statistically significant differences in the other eight outcome measures, including ADL dependence, behaviours, caregiver self-efficacy, and upset scores between the experimental and control groups - Analyses showed a trend toward improvement in all areas for the experimental group, but these minimal effects were not statistically significant Psychological Wellbeing Outcomes: - Women reported enhanced self-efficacy in managing behaviours (*p* = 0.038) Both women (*p* = 0.049) and minorities (*p* = 0.037) reported enhanced self-efficacy in managing functional dependency	- Interventionists reported that caregivers who initially rejected recommendations often inquired about these strategies at the final intervention visit - A consistent finding in research on the use of environmental modifications is that individuals are highly selective in their acceptance and use of strategies and need repeated opportunities to think about and practice strategies The data suggest that this approach is helpful for female, African American, and spouse caregivers, but the intervention would need adjustments to meet the needs of male and no spouse caregivers	2
Kelley et al. (2019); UK [35]	Qualitative ethnographic design. Data collection methods included participant observations, informal conversations, and in-depth semi-structured interviews	Two carers of older people acute hospital wards in two cities in the north of England Specific settings: An 18-bedded rehabilitation ward A 24-bedded general hospital ward	n = 12 people with dementia were purposefully sampled; relatives and friends included 8 daughters, 2 husbands, 2 sons, 2 granddaughters, 1 wife, and 1 friend; hospital staff: n = 23, including doctors, nurses, healthcare assistants, and therapists (e.g., physiotherapists and occupational therapists)	To explore the involvement of families in the hospital care of people living with dementia	Patient-Oriented Research: Observational ethnographic approach involving participant observations, informal conversations, and semi-structured interviews Direct Family Engagement: Families provided critical input on patient care routines, preferences, and communication methods - Families directly participated in aspects such as dressing, feeding, and routine personalization	Screening: Families were indirectly involved in screening and assessments by providing background knowledge and observations on the patient’s condition Example: Families could hold crucial information for interpreting the needs of people living with dementia, families’ input in hospital records highlighted potential health deviations Treatment Plan Development: Family contributions included sharing patient preferences and routines, which informed treatment adjustments Information from families could help maintain connections to usual routines by informing personalisation of care routines Treatment Plan Implementation: Families were actively involved in care delivery, especially for activities like feeding, dressing, and encouraging participation in therapy tasks Example: some families directly helped administer medications when staff struggled Rehabilitation: Families played a role in rehabilitation by engaging patients in familiar routines or activities that supported recovery, such as playing games or continuing hobbies like photography Tools Used: - No specific mention of formal tools used by families, but their input included communication aids like personal stories, familiar items, and photographs that facilitated care and connection	Demographics: - People with Dementia: Gender: 5 men, 7 women Stage of Dementia: ranged from suspected early stages to advanced diagnosed dementia Pre-admission Living Arrangements: 11 lived at home or in sheltered housing; 1 was living in a care home Length of Stay: varied from 13 to 78 days (median 24 days) Discharge Destinations: 4 returned homes with increased support, 7 were discharged to a care home, and 1 died before discharge - Family and Friends: Relationships: 8 daughters, 2 sons, 2 husbands, 1 wife, 2 granddaughters, and 1 friend Visiting Frequency: ranged from daily to a few times per week Pre-hospital Support: varied from occasional tasks (e.g., shopping, meals) to intensive daily care (e.g., washing, dressing) Health Problems Dealt With: dementia-related complications such as confusion, delirium, infections, falls, fractures, and suspected strokes Physical Wellbeing Outcomes - Outcomes of Hospitalization: Prolonged disconnection from usual routines exacerbated confusion and led to loss of abilities Lack of personalization in care led to reduced food and drink intake and increased risks of falls Patients discharged often required new or increased care support, and several transitioned to care homes permanently - Impact of Family Involvement: Families provided critical information to identify health changes, such as infections, by recognizing behavioural changes like agitation or reduced appetite Personalized engagement by families, such as dressing or feeding assistance, improved patient comfort and reduced distress	Positive Health Outcomes: Families’ knowledge could also help staff engage people living with dementia in activities such as assessments and therapy tasks, leading to better participation and outcomes Families recognized signs that their relative was more unwell or in need, even when the person had significant communication difficulties Non-Statistically Significant Outcomes: Negative Health Impacts: Prolonged disruptions to familiar routines and levels of functioning could exacerbate confusion and cause the person to lose, through lack of practice, connections to valuable abilities Irreversible functional decline could lead to increased care post-discharge, including residential care, causing further disconnections from previous life Psychological Wellbeing Outcomes: Positive Psychological Outcomes: Families’ involvement often created a sense of comfort for people living with dementia, lessening the unfamiliarity of ward environments and routines Maintaining family connections during hospitalization was a key concern for people living with dementia, many of whom attached great value to these relationships and the opportunities visiting times offered to maintain them People who were distressed or anxious had a particularly high need for connection with others. Family involvement helped alleviate fear and distress Negative Psychological Outcomes: A lack of interaction and stimulating features in hospital environments could lead to feelings of disorientation, distress, and confusion A lack of staff presence or time to interact was linked to an increased risk of agitation and falls	Satisfaction of Family Members Improved Satisfaction: Families spoke positively about being able to contribute to care by providing personal items or helping with daily tasks like feeding and dressing, which allowed them to feel involved and reassured about the quality of care Families appreciated staff efforts to include them in conversations about care routines and discharge planning Dissatisfaction: Families sometimes expressed frustration with restricted visiting hours and the lack of clear guidelines about how they could contribute to care - Some families were dissatisfied with staff interactions when they perceived them as hurried or inattentive to patient needs	5
Black et al., (2013); USA [36]	Cross-sectional	Urban, Maryland	Community-residing people with dementia (PWD): n = 254 Informal caregivers: n = 246	The study emphasizes the importance of family members and friends in the care of community-residing individuals with dementia	Integration was achieved through various caregiver tasks, including assisting with ADLs, managing safety and behavioural symptoms, coordinating services, facilitating health care visits, and advocating for the individual with dementia	- Screening: Families were involved in early diagnosis which is crucial for planning future care - Treatment Plan Implementation: Caregivers were actively involved in various aspects of care, including ADLs, safety management, and coordination of services - Rehabilitation: The study addresses the correlation between symptoms of depression and unmet needs, indicating a focus on mental health - Tools Used: The Johns Hopkins Dementia Care Needs Assessment (JHDCNA) was used for comprehensive assessment, covering multiple domains relevant to dementia care	Demographics: community-residing persons with dementia (PWD): n = 254; informal caregivers: n = 246 Health Issues Dealt With: dementia	Statistically Significant Outcomes: - Unmet safety needs: 90% of PWD had unmet safety needs, particularly related to fall risk and wander risk management and home safety evaluations - Cognitive function: unmet needs in PWD were significantly greater among those with higher cognitive function - Depression and unmet needs: symptoms of depression were correlated with more unmet needs in both caregivers and care recipients Psychological Wellbeing Outcomes: Cognitive function and unmet needs: unmet needs in PWD were significantly greater among those with higher cognitive function	Unmet needs in caregivers: over 85% of caregivers had unmet needs for referrals to community resources and caregiver education on topics such as how dementia affects individuals and their loved ones, availability of community-based services, caregiver skills Mental health domain: 45% of caregivers had unmet needs in the mental health domain, with most requiring emotional support or respite care	2
Carnevale et al., (2002); USA [37]	Repeated measures randomized design with three groups (control, education only, education plus behaviour management)	Community settings in northeastern New Jersey	Persons with brain injury with their caregiver: n = 27 each	To address unmet caregiver needs and manage caregiver burden associated with TBI-related neurobehavioral challenges	Community-delivered behaviour management program (NSBM) with educational and interventional modules	- Screening: Initial screening involved a brief telephone interview followed by an intake assessment in the community setting. Caregivers were engaged in identifying target behaviours and developing behavioural assessment procedures - Treatment Plan Development: Caregivers were actively involved in developing individualized treatment plans. This process included identifying target behaviours, discussing intervention strategies, and providing feedback - Treatment Plan Implementation: Caregivers were responsible for implementing intervention strategies independently and reporting progress in follow-up sessions - Rehabilitation: The study emphasized long-term support and autonomy for patients and caregivers, integrating rehabilitation efforts within the community setting - Tools Used: For Screening and Assessment: Questionnaire on Resources and Stress (QRS), Maslach Burnout Inventory (MBI). - For Treatment Implementation: behavioural assessment procedures, video-taped observations, and clinical vignettes	Demographics: - The final sample consisted of 27 adult patients with brain injuries: 18 men and 9 women; 18 patients had traumatic brain injuries (TBIs) from various causes: 11 motor vehicle accidents, 3 pedestrians hit by motor vehicles, 2 bicyclists hit by motor vehicles, 1 construction-related accident, 1 assault; 9 patients had non-traumatic brain injuries: 5 anoxia cases, 1 arteriovenous malformation, 1 stroke, 1 encephalopathy, 1 electrocution - Mean age of patients: 38.9 years old (SD 11.5) - Mean age of caregivers: 47.5 years old (SD 14.4) - There were no statistically significant differences between groups in terms of patient age (F = 0.58, *p* > 0.05) or caregiver age (F = 1.63, *p* > 0.05) - The sample was predominantly Caucasian (85.2%) - Most patients were high school graduates (37%) or had partial college/trade school training (25.9%) Health Problems Dealt With: Traumatic brain injuries (TBIs) from various causes (motor vehicle accidents, pedestrian accidents, bicycle accidents, construction-related accidents, assaults)	Statistically Significant Outcomes: - Emotional exhaustion (EE): dependent variable; MBI scale emotional exhaustion (EE); corrected model: F(3, 22) = 22.631, *p* < 0.000; covariate: F(1, 22) = 64.383, *p* < 0.000; adjusted R squared: 0.722 (indicating a strong model fit) - Depersonalization (DP): dependent variable; MBI scale depersonalization (DP); corrected model: F(3, 23) = 11.130, *p* < 0.000; covariate: F(1, 23) = 32.455, *p* < 0.000; adjusted R squared: 0.539 Non-Statistically Significant Outcomes: - QRS subscales (Limits on Family Opportunities, Pessimism, Personal Burden for Respondent): for all QRS subscales, after adjustment for baseline levels, the outcome after treatment was not statistically significant - MBI subscales (EE, DP, PA): after adjustment for baseline levels, the outcomes for MBI subscales were not statistically significant Psychological Wellbeing Outcomes: - Pessimism: dependent variable; QRS scale pessimism; corrected model: F(3, 20) = 3.102, *p* = 0.050; covariate: F(1, 20) = 5.733, *p* = 0.027; adjusted R squared: 0.215 Personal burden: dependent variable; QRS scale personal burden; corrected model: F(3, 19) = 2.829, *p* = 0.066; covariate: F(1, 19) = 8.355, *p* = 0.009; adjusted R squared: 0.200	- The study examined the impact of an intensive community-based behaviour management program on caregivers’ stress and burden. A major finding was that initial levels of caregivers’ burden and distress were highly predictive of their ratings at outcome, irrespective of participation in treatment	2
Woods et al., (2016); United Kingdom [38]	Pragmatic multi-centre randomised controlled trial	Community settings, primarily in urban and regional areas	Total days that completed the study: 350	To assess the effectiveness and cost-effectiveness of joint reminiscence groups	Patient-oriented research involving joint reminiscence groups for people with dementia and their carers	Screening or Assessment: Families were involved as informants in the clinical assessment of participants’ eligibility (e.g., DSM-IV criteria for dementia) Treatment Plan Development: Each session blended work in large and small groups, and a range of activities including art, cooking, physical re-enactment of memories, singing and oral reminiscence Treatment Plan Implementation: Carers participated directly in delivering the intervention by engaging in group sessions with their family members Carers were guided to value their contribution by allowing the person with dementia to actively participate Tools Used: Clifton Assessment Procedures for the Elderly (used in clinical screening for dementia) - General Health Questionnaire (GHQ-28), Quality of Life in Alzheimer’s Disease (QoL-AD), and other scales used for outcome measurement	Demographics: Of the people with dementia, 242 (50%) were female, 447 (95%) were of white ethnicity, 337 (72%) were married, and mean age was 77.5 (SD 7.3) The carers comprised 325 (67%) females, 448 (96%) of white ethnicity, 345 (74%) were caring for their spouse, and mean age was 69.7 (SD 11.6) Health Problems Dealt With: Focused on mild to moderate dementia diagnosed using DSM-IV criteria	Statistically Significant Outcomes: Increased anxiety in carers at the ten-month endpoint was statistically significant Carers of people with dementia allocated to the reminiscence intervention had significantly increased anxiety on the General Health Questionnaire-28 sub-scale at the ten-month endpoint (mean difference 1.25, F = 8.28, *p* = 0.04) Non-Statistically Significant Outcomes: No significant differences in primary outcomes (e.g., quality of life for the person with dementia, carer mental health) between intervention and control groups The intention to treat analyses indicated there were no differences in outcome between the intervention and control conditions on primary outcomes at the ten-month endpoint (self-reported QoL-AD mean difference 0.07, F = 0.48, *p* = 0.53) No significant differences in secondary outcomes like daily living activities, depression, or relationship quality There were no significant differences in secondary outcome measures, at either ten or three-month endpoints Psychological Wellbeing Outcomes Limited psychological benefits for carers Carers showed increased stress related to caregiving associated with more sessions attended Some benefits for people with dementia who attended sessions Compliance analyses suggested improved autobiographical memory, quality of life, and relationship quality for people with dementia attending more reminiscence sessions	Satisfaction of Family Members: Of 215 participants responding to a feedback survey at the end of the weekly groups, all but 3 said that they would recommend the program to a friend Families reported enjoyment despite lack of clinical improvement - The lack of positive findings stands in contrast to the reported enjoyment and benefits reported by participants and facilitators	2
Schoenmakers et al., (2010); Belgium [39]	Randomized controlled trial (RCT), quasi design; interviews; psychometric testing	Community-dwelling elder in a mixed rural-city area of Leuven, Belgium	Initial screening: 346 Included in the trial: 62 Follow-up interviews after six months: 52 Follow-up interviews after twelve months: 46	Intervention of a care counsellor, coordinating home care in a non-hierarchical and quasi-unstructured way for one year, will alleviate carers’ feelings of depression	- Monthly phone calls, three-monthly visits to inventory care needs, organizing formal support, and continuous availability of a care counsellor over the course of one year - The care counsellor guided the family carer in organizing home care and assisted the family carer in exploring any problematic home care situations	Screening: Tools used: Mini Mental State Examination (MMSE), Zarit Burden Inventory, Ways of Coping Checklist, Stai-instrument for anxiety Treatment Plan Development: - During the ongoing of the study and in particular with each new intervention, the care counsellor was supervised and given feedback by a skilled general practitioner. The care counsellor was asked to write down an unstructured report on every provided and extra contact with the carer - General practitioners were informed about each change in formal or informal home care of their patients Treatment Plan Implementation: - Ten carers applied for extra help. Four of these carers were satisfied with an extra visit of the care counsellor. A new intervention was proposed to six carers. In two cases, day care was introduced, two personal alarms, and one offer for extra in-home help were proposed. Three proposals (two alarms, one day-care stay) were effectively carried out	- There were 120 female caregivers (58%) and 86 male caregivers (42%) in the study - Most caregivers were white (90%) - Age distribution: 86% were at least 60 years old at baseline; more than half (59%) were at least 70 years old - Cognitive impairment (GDS Scores): 32% of the patients had moderate cognitive impairment (GDS = 4); 40% had moderately severe impairment (GDS = 5); 28% had severe impairment (GDS = 6) - Income: 86% of patients had annual incomes less than USD 25,000; 51% had incomes less than USD 10,000; more than half of caregivers (60.2%) reported being worried about their financial futures Health Problems Dealt With: Cognitive decline; mood swings and disruptive behaviour (e.g., screaming, aggression)	Statistically Significant Outcomes: Depression: one year after the start of the intervention, the odds ratio for depression in the treatment group versus the control group was 0.16 (95% CI 0.03–0.86); sequential analysis showed no significant change in the odds ratio for depression when considering gender and relationship status Psychological Wellbeing Outcomes for Patients: - Patients exhibited moderate to high frailty and were limited in performing instrumental tasks of daily living - Cognitive decline: patients showed mild-to-moderate cognitive decline - Continence problems: three-quarters of patients faced continence issues - Mood swings and disruptive behaviour (e.g., screaming, aggression) were present in one-third of the patients	- The actual intervention of the care counsellor was described as minimal - Ten carers applied for extra support, but only one carer called the care counsellor outside the provided appointments - Only half of the proposed interventions were effectively carried out	2
Manning et al., (2020); USA [40]	Experimental study; interview; psychometric test	NA	105 people with dementia and their caregivers	To address the increased morbidity and poorer health among caregivers and to mitigate negative biopsychosocial impacts and prevent institutional placements	Care coordination involves education, support, guidance, and access to resources	Treatment plan development: Families received guidance and support but not specifically involved in co-designing services	Demographics: participants included 105 people with dementia, of whom 54.3% were women; 67.3% of the caregivers were women Health Problems Dealt With: Dementia	Statistically Significant Outcomes: - There were significant decreases in caregiver burden and depression over time (*p* < 0.001 for both) - Regarding healthcare utilization, there was a decrease in the number of nights spent in the hospital with care coordination, compared to the period before the study onset - APIM results showed that patients’ and caregivers’ depression at baseline were positively correlated (*p* < 0.001) - Patients’ depression at baseline was a predictor of their depression at 12 months (*p* < 0.01), taking into account the correlation between patient and caregiver depression, as well as the effect of caregivers’ depression at baseline Psychological Wellbeing Outcomes: - Care coordination may help alleviate the negative impacts related to caregiving, suggesting this model could improve important elements of dementia care	- Care coordination satisfaction was reported to be high - Patients and caregivers participating in a novel caregiving model that involved care coordination showed decreased caregiver burden, depression, hospitalization	2
Lennaerts et al., (2017); Netherlands [41]	Mixed methods study, including: - Explorative qualitative after-death interviews with bereaved family caregivers and professionals - Multiple-case study incorporating in-depth interviews and questionnaires	Hospitals General practices Nursing homes Focused coverage via ParkinsonNet in urban and regional settings	5–15 patients with advanced Parkinson’s disease and their family caregivers, bereaved family caregivers, and 10 professionals from various disciplines	To explore patients’ unmet needs and involvement in care provision	Patient-oriented research using interviews and questionnaires	Screening Involvement: Patients will be included based on an affirmative answer from the attending physician on the so-called ‘surprise question’ Family caregivers will also complete questionnaires like the Zarit Burden Interview (ZBI) to assess caregiver burden Assessment Tools: Zarit Burden Interview (ZBI): used for assessing caregiver burden Edmonton Symptom Assessment Scale (ESAS-PD): used for patient symptoms but involved family caregivers for corroboration Treatment Plan Development: - Data collected during interviews and questionnaires will inform palliative care protocols and service improvements Treatment Plan Implementation: Caregivers supported patients during interviews and provided information about their needs	1. Demographics: The study collected comprehensive socio-demographic data for all participants, ensuring diversity in experiences. Participants included patients, family caregivers, and bereaved caregivers, primarily from the Netherlands Participants include 5–15 patients with advanced Parkinson’s disease and their family caregivers, bereaved family caregivers, and 10 professionals from various disciplines 2. Health Problems Addressed: Parkinson’s disease, targeting symptoms, such as motor difficulties, cognitive impairments, psychological symptoms (e.g., depression, hallucinations), and caregiver burden 3. Physical Wellbeing Outcomes: Outcomes were evaluated using validated tools like ESAS-PD and FACT-G to assess physical symptoms and functional wellbeing, alongside quality-of-life questionnaires tailored to Parkinson’s disease	Positive Health Outcomes: - The study used various validated scales to assess outcomes related to mobility, independence, symptom management, and functional wellbeing Psychological Wellbeing Outcomes - Positive psychological outcomes: the Parkinson Disease Questionnaire 8 (PDQ-8) captures perceptions of illness, which include psychological dimensions of health Caregivers often felt emotional strain due to the demands of advanced PD care and reported this in interviews - Negative psychological outcomes: family caregivers experienced pre-death grief, associated significantly with the cognitive decline of patients	Caregiver Satisfaction: Caregivers expressed a lack of satisfaction due to poor coordination of palliative care services	4
Livingston et al., (2020); UK [42]	Randomized controlled trial (RCT). It is a parallel-group, single-blind superiority trial	The study was conducted across four sites in the UK: A large city mental health trust A semi-rural area trust A tertiary neurological clinic for rare and young-onset dementia A mental health trust with specialist nurse services (admiral nurses)	Total participants: 260 family carers Intervention group: 173 carers Control group: 87 carers (treatment as usual) Participants were self-identified family carers of individuals diagnosed with dementia	To evaluate the effectiveness of the START (STrAtegies for RelaTives) intervention over six years, aiming to alleviate their anxiety and depressive symptoms	The START multicomponent intervention for family carers is individually delivered by supervised psychology graduates (with a first degree in psychology and no clinical training) and was tested by our research team in a randomised controlled trial (RCT) Therapists worked with carers to identify individual difficulties and implement strategies including behavioural management, communication strategies, identifying and changing unhelpful thoughts, positive reframing, accessing support, future planning, and increasing pleasant events	Screening: We collected carer and patient sociodemographic details at baseline and measured dementia severity using the Clinical Dementia Rating We also administered the Neuropsychiatric Inventory (NPI), as neuropsychiatric symptoms have been shown to be associated with carer psychological morbidity and the Zarit Burden Interview Treatment Plan Development: Each session included a relaxation exercise, and we asked carers to put into practice the individualized strategies and relaxation between sessions. The final session was used to agree on a plan of what to do in the future based upon what that carer had felt worked Patients in both groups received TAU (treatment as usual) and the use of services in both groups has been described in detail Treatment Plan Implementation: Therapists worked with carers to implement strategies including behavioural management, communication strategies, and positive reframing Tools Used: Screening tools: Clinical Dementia Rating, Neuropsychiatric Inventory (NPI), Zarit Burden Interview, Hospital Anxiety and Depression Scale (HADS)	Demographics: We recruited 260 participants to study Carers were mostly spouses/partners (109; 42%) or children (113; 44%) Health Problems Dealt With: Focus on dementia’s impact, including carers’ mental health challenges such as anxiety and depression Physical Wellbeing Outcomes: Time spent at home (median time until death or admission to care): START group = 42.2 months vs. TAU group = 39.0 months, though not statistically significant There were no significant group differences in patient-related physical health outcomes, such as time to care home admission or death rates	Statistically Significant Health Outcomes: Analysis of HADS-T, adjusting for centre, baseline score, time, and factors related to outcome (carer age and gender, NPI, Zarit) over the 6-year period, showed an average improvement in HADS-T of 2.00 points compared with TAU (95% CI −3.38 to −0.63; *p* = 0.005) Fully adjusted models for HADS-D and HADS-A continuous scores indicated significant beneficial intervention effects over 6 years, with average decreases of −1.06 (95% CI −1.78 to −0.35) for depression and −0.97 (95% CI −1.78 to −0.15) for anxiety Non-Statistically Significant Health Outcomes: Reduction in HADS-anxiety cases, however, was not significant (OR = 0.50, 95% CI: 0.24–1.07, *p* = 0.07) Time until care home admission or death showed no significant between-group differences (Intensity Ratio 0.88, 95% CI 0.58–1.35) Psychological Wellbeing Outcomes: The START intervention reduced depressive and anxiety symptoms of family carers of relatives with dementia at home over 2 years and was cost-effective In the intervention group, carers were five times less likely to have clinically significant depression on a validated rating scale Specific Observations: Improvements in anxiety and depression scores for family carers as measured by HADS (Hospital Anxiety and Depression Scale) Sustained improvements over six years in depression and anxiety outcomes for family carers	Carers reported satisfaction with the intervention, emphasizing its role in providing practical coping mechanisms and improved quality of life	2

## 3. Results

### 3.1. The Study Characteristics

**Figure 1:**
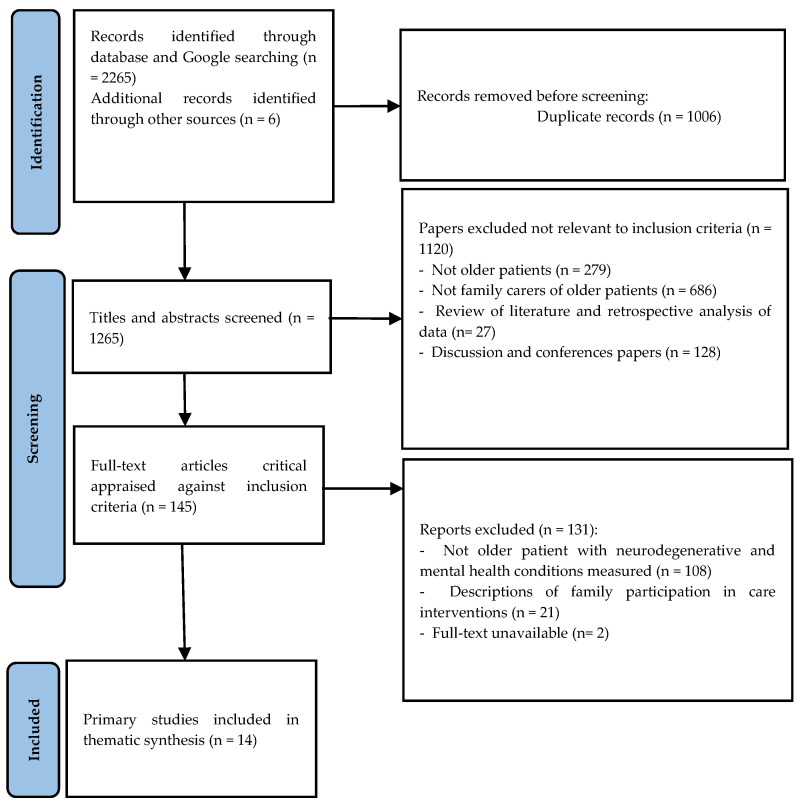
PRISMA 2020 flow diagram of the selection process for studies.

Fourteen studies were reviewed as illustrated in Figure 1. All studies originated from high-income countries, with the majority conducted in the USA (*n* = 7) and the UK (*n* = 5), and additional studies from Belgium (*n* = 1) and the Netherlands (*n* = 1). The timeline of these publications shows a growing body of evidence on family integration in health service delivery for OPNMHCs over the last two decades. Among the papers, six studies used randomised controlled trials (RCTS) to assess the impact of family-integrated care models. Three studies were qualitative, two employed mixed methods approaches, and three utilised psychometric testing in conjunction with quasi-experimental or observational designs. Data collection encompassed input from OPNMHCs, family carers, and healthcare professionals to evaluate health and services outcomes and satisfaction with family-integrated care.

Most family-integrated care models were implemented in community settings, including home-based care (*n* = 10), followed by hospitals (*n* = 4) and rehabilitation clinics (*n* = 2). Studies examined the impact of family-integrated care models for various neurodegenerative and mental health conditions, including Alzheimer’s and dementia (*n* = 6), Parkinson’s disease (*n* = 2), cognitive decline (*n* = 2), traumatic brain injury (*n* = 1), depression (*n* = 4), and anxiety (*n* = 3). The mean age of OPNMHCs reported in nine studies ranged from 65 years to 80 years, with samples predominantly recruited from females. The family carers involved in care interventions were primarily spouses (*n* = 12), children (*n* = 10), and other relatives or friends (*n* = 5), aged between 46 and 65 years. The duration of family integration in the interventions varied, ranging from short-term interventions (up to 6 months; *n* = 9) to long-term programs (over 6 months; *n* = 5).

### 3.2. An Overview of the Fourteen Included Studies Is Provided in Table 3

A synthesis of this detailed data reveals several overarching patterns. Interventions conducted in community settings consistently reported positive outcomes related to caregiver satisfaction, reduced caregiver burden, and improved patient functional status. In contrast, hospital-based models, while beneficial, showed more variable results, particularly regarding mental health outcomes for patients with dementia. Statistically significant improvements in patient or carer outcomes were most frequently reported in studies with longer intervention durations (six months or more) and those that incorporated structured, goal-oriented caregiver education and training.

### 3.3. Identified Themes

In addition to the study characteristics, the synthesis of the study findings generated the following four key themes: family participation in service delivery, patient health and wellbeing outcomes, satisfaction with care, and service dynamics that enable successful family-integrated care. Among the studies reviewed, a noticeable trend emerged showing that family-integrated care models were more successful in community-based settings, particularly in improving patient mobility and caregiver satisfaction. Hospital-based models, on the other hand, demonstrated more varied results in improving mental health outcomes, particularly for patients with dementia.

#### 3.3.1. Family Participation in Service Delivery

The integration of family carers occurred across various health services’ delivery to OPNMHCs, including goal setting [29,30], co-designing care plans [29,31], decision making in patient care [30,31,32,33,34,35], and assisting with direct care [30,32,34,35,36]. Studies reported that direct care tasks performed by the family carers involved patient assessment and providing physical and non-physical care during treatments and the rehabilitation of the patients [30,32,35,36]. Screening and clinical assessments of older patients at risk of neurodegenerative and mental health conditions involved family carers, with a focus on behavioural challenges [29,37] and functional independence [34]. Family carers were also involved in care coordination [29,30,31,36], providing input in medication review [29], advocating for patient preferences [29,30,32,33], assisting with daily living activities like preparing meals, hygiene maintenance, mobility support, managing behavioural symptoms, and providing physical and emotional support [30,32,34,35,36]. In addition to the direct care tasks, family carers were involved in group interventions. Examples included carer–patient joint reminiscence groups [38], functional recovery support [30], and home-based physical therapy [29]. Rehabilitation services, such as behavioural management programs and goal-attainment scaling, included family carers in multiple interventions [33,37].

#### 3.3.2. Patient Health and Wellbeing Outcomes

While the focus of family-integrated care models varied across healthcare settings, these models demonstrated positive psychological and physical health outcomes for OPNMHCs [29,30,32,33,34,35,36,37,38,39]. In implementing community-based interventions, Gitlin et al. [32] aimed to reduce agitation and behavioural disturbances among OPNMHCs, while Fortinsky et al. [29] targeted a 25% decrease in emergency department visits and hospitalisations for patients living with dementia through family-integrated care delivery. Conversely, hospital-based interventions primarily focused on functional recovery and mobility improvements. For example, Boltz et al. [30] focused on improving activities of daily living (ADL) and functional independence among patients who received Fam-FFC intervention in hospital settings. Eight studies reported improvements in patient mental health outcomes following family-integrated interventions [30,31,32,33,34,38,39,40,41,42]. Significant reductions were noted in anxiety and depressive symptoms among OPNMHCs [29,30,40]. However, improvements in quality of life and social engagement were non-significant [30,31,34]. Regarding functional outcomes, significant improvements were demonstrated in activities of daily living (ADL), condition severity, and walking performance of OPNMHCs [30,38]. In addition, interventions lasting between 6 and 12 months demonstrated a more sustained impact on patient functional outcomes [30,32,39].

#### 3.3.3. Satisfaction with Care Involving Family Members

Ten studies reported satisfaction with family-integrated care coordination and intervention programs among different participant groups, including OPNMHCs [36], family carers [32,34,35,36,37,38,39,40], and healthcare professionals [29,30]. OPNMHCs who benefited from family-integrated care models shared their experience of enhanced comfort and emotional reassurance due to family involvement [36]. Satisfaction among family carers was consistently reported, with notable improvements in preparedness for caregiving [30,40], decreased caregiver burden and depression [29,40], and an alignment of interventions with personalised caregiving goals [33]. Family carers particularly valued improvements in their coping abilities, quality of life [42], and effectiveness in managing the mood and behaviour of OPNMHCs [32]. Family carers expressed comfort and satisfaction with trained interventionists [29,32] and appreciated structured processes that facilitated goal attainment and the management of repetitive behaviours [33]. Healthcare professionals responded positively to these care models, valuing family carer involvement, as it enabled more comprehensive and personalised care, as well as effective care delivery [32]. Positive attitudes between family carers and healthcare professionals during the interventions fostered collaboration and mutual respect, further enhancing the quality of care for OPNMHCs [35].

#### 3.3.4. Service Dynamics in Enabling Family-Integrated Care to Be Successful

Family carers co-designed intervention strategies and participated directly in service delivery for OPNMHCs [29,30,31,32,33,34,36,38]. Structured discussions and goal setting with family carers were central features in eight intervention programs, including activities such as developing behaviour management plans [32,37] and facilitating a reminiscence group [38]. Improved care coordination, driven by family carers’ practical experience, personalised one-to-one support, extended involvement times [29,30,32], additional structured sessions [33], active listening, motivational interactions [35], and medication management support [29], and led to positive health and wellbeing outcomes for OPNMHCs. Furthermore, family carers’ involvement expanded the scope and quality of care, creating a sense of belonging and enhancing the OPNMHCs’ perceptions of being well cared for [30,32,35,37,38,39,40,41].

Incorporating family carers into healthcare practices was instrumental in improving quality of care and facilitating patient connections to community resources [36]. This collaboration assisted hospital staff in preparing patients for earlier discharge, providing staff with flexible care strategies and valuable experiential learning opportunities through interactions with family members [29,30,31,33,34,35,37,38,39,40,41]. Moreover, the direct engagement of family carers in screening and the development and implementation of treatment plans enhanced care delivery outcomes for OPNMHCs and boosted their self-efficacy in managing patients.

## 4. Discussion and Conclusions

This review of family-integrated care models for OPNMHCs demonstrates that integrating family carers into the assessment and management of care can lead to improved patient health outcomes and increased satisfaction with care across acute and primary care settings. The analysis of diverse integration strategies suggests that family involvement, whether through goal setting, direct care provision, or care coordination, can enhance the accessibility and effectiveness of healthcare services for OPNMHCS. The evidence synthesis reveals that family-integrated care models work more effectively in community-based settings where family carers are continuously involved in care delivery. In contrast, hospital-based models tend to focus more on short-term goals like crisis management, resulting in more variable outcomes for patients’ psychological and functional recovery.

The reviewed studies consistently reported positive perceptions of family integration among all stakeholders, including OPNMHCs, family carers, and healthcare professionals. Findings from both qualitative and quantitative studies confirmed that family-integrated care was associated with favourable experiences for OPNMHCs, particularly in terms of patient engagement and service usage. Although the evidence base for the impact of family-integrated care models on the health outcomes and care satisfaction of OPNMHCs remains limited, the existing literature indicates promising trends. Variability in integration approaches and outcome measures across studies makes it difficult to draw definitive conclusions; however, a general pattern of benefit emerges. Many reviewed models emphasised person-centred care principles, demonstrating improvements in neuropathic symptoms, functional capacity, and overall quality of life. Over time, studies have consistently shown positive psychological and functional outcomes for OPNMHCs due to family engagement in care delivery [16,17,20].

Family carers were found to be key contributors across all interventions, actively supporting both short-term needs and long-term recovery goals. These findings suggest a shift in care models—from focusing solely on clinical outcomes to incorporating more holistic elements, such as group-based psychosocial interventions. This broader approach holds significant implications for how services are designed and delivered in the management of neurodegenerative and mental health conditions. Furthermore, while implementing recovery-oriented or trauma-informed care remains challenging in acute and primary care settings, the structured inclusion of family carers offers a pathway to more responsive and satisfactory healthcare experiences.

There were some variations in how family carers were involved in designing and delivering services for OPNMHCs. Across the reviewed studies, family integration ranged from minimal involvement in routine care tasks to active participation in collaborative care planning and shared decision-making processes [43]. Family integration in service delivery differs based on disease and patient needs. Additionally, in some cases, family carers are considered as adjunct to the core tasks of delivering services to patients [44,45]. However, it is evident that in models where carers were included in setting goals, contributing to care plans, and participating in treatment decisions, the care delivery was more aligned with person-centred principles, leading to better engagement and outcomes for patients. Evidence suggests that such involvement enhances the relevance and responsiveness of care to the individual’s needs and improves service utilisation and continuity of care [17,20]. Finally, variations in the length of family integration in care models may also have affected how family carers are integrated in service design and delivery, as shorter interventions tend to favour the ‘one-off’ type activities, such as screening or goal setting.

Despite the well-documented benefits of family-integrated care models, several challenges hinder their effective and sustainable implementation. Existing evidence highlights barriers such as insufficient resources for family carers, unmet support needs, caregiving burden, and a general lack of preparedness among carers. Studies by Aung et al. [46], Malmir et al. [47], and Rahmani et al. [48] showed that without adequate financial, emotional, and institutional support, family carers often struggle to fulfil their roles effectively. Black et al. [36], Carnevale et al. [37], and Schoenmakers et al. [39] similarly underscore how limited access to resources and formal support services can restrict meaningful family engagement in care planning and delivery. Although some educational and training interventions have demonstrated short-term improvements in carer knowledge, confidence, and competence, the long-term sustainability of these outcomes remains uncertain. Schoenmakers et al. [39] reported measurable improvements in care outcomes following caregiver education but also expressed concerns about the durability of these effects, particularly in the absence of ongoing support and follow-up. Addressing these challenges requires initial investments in caregiver training and continuous support mechanisms, system-level resource allocation, and structured follow-up to maintain caregiver engagement and prevent burnout.

Family-integrated care models show promise, but at the same time, several systemic barriers exist, including a lack of formal training for caregivers, limited resources, and institutional resistance to change. Effective partnerships between family caregivers and healthcare professionals are critical to the management of neurodegenerative and mental health conditions. Several studies have noted that healthcare professionals do not always value the contributions of family carers in service design and delivery. For instance, Wolff et al. [49] and Hazzan et al. [50] found that healthcare professionals often restrict the role of family carers to basic care tasks, rather than involving them in the broader management of dementia and depression. A key barrier to building strong partnerships is the lack of time, consistently identified by family carers and healthcare providers [50]. Compounding this issue is the healthcare system’s emphasis on medical outcomes, which can limit opportunities for more holistic, person-centred care and collaboration. Recognising family carers as partners in care and acknowledging their contribution can help address these challenges. Initiatives such as appointing family resource champions or implementing care coordination programs may offer practical ways to support healthcare professionals in fostering open communication and stronger partnerships with family carers.

The methodological heterogeneity of the included studies is a crucial consideration when interpreting the findings of this review. By synthesising evidence from RCTs, qualitative studies, and other designs, our review provides a broad perspective. Still, it is limited in its ability to make strong causal inferences or to quantify the precise effect of family-integrated care. The inclusion of diverse study designs precluded the use of any form of meta-analysis. Our thematic synthesis approach was, therefore, chosen as an appropriate method to identify common patterns and generate insights across this varied evidence base. The MMAT was used to systematically assess quality, which was considered during synthesis; however, we did not exclude studies based on low quality scores, choosing instead to synthesise the full spectrum of available evidence. Consequently, the findings should be interpreted as a descriptive summary of the current state of the field, highlighting promising trends rather than establishing definitive evidence of effectiveness.

Gaps exist in the literature regarding the processes and economic implications of integrating family carers into healthcare services design and delivery for older patients, including those with neurodegenerative and mental health conditions (OPNMHCS). Although available studies increasingly support the involvement of family carers in both community and hospital-based care, most studies have concentrated on measuring health and service outcomes, such as improvements in activities of daily living, mobility, emotional wellbeing, and reductions in hospital admissions and readmissions. In contrast, there is limited exploration of process-oriented dimensions, including how health systems accept and adopt family integration, its cost-effectiveness, and long-term sustainability. Future research should incorporate comprehensive economic analyses and sustainability evaluations to address this gap. Such a holistic approach is important to realise the clinical benefits and the enduring feasibility and value of embedding family involvement in the care of OPNMHCS.

This review is limited to the selected neurodegenerative and mental health conditions and each author’s interpretation of the study results. In addition, the number of studies was limited, and the methodologies employed in the reviewed studies were often not designed to demonstrate significant causal associations between family integration and health outcomes for OPNMHCs but instead reported on relationships between the two. This review’s inclusion criteria focused exclusively on studies describing the family-integrated care models, excluding some studies that fell outside this criterion. A few studies were excluded, as the family-integrated care models were not discussed in detail, reducing the number of studies for this review. Furthermore, the restriction to English-language publications may have introduced a language bias, potentially excluding relevant studies from non-English-speaking regions where cultural contexts and healthcare systems influencing family caregiving may differ significantly.

Future research should aim to explore the long-term sustainability of family-integrated care interventions. Longitudinal studies with larger sample sizes are needed to assess the lasting impact of family involvement on patient outcomes and caregiver wellbeing. Additionally, economic evaluations of family-integrated care models are necessary to determine their cost-effectiveness and feasibility in primary and hospital care settings.

### Implications for Policy and Clinical Practice

The findings of this review have important implications for clinical practice for treating neurodegenerative and mental health conditions. While international and Australian frameworks encourage family partnership in aged care [14,15], this review highlights a lack of standardised models specifically for OPNMHCs. To bridge this gap, future policy and service design should focus on formally recognising, resourcing, and standardising the involvement of family carers. Based on the evidence synthesised, effective frameworks should be built on several core principles, as follows:

- Frameworks must mandate a collaborative approach that treats family carers as essential partners in care. This includes structured processes for their involvement in goal setting, care planning, and decision making, moving beyond peripheral tasks.
- To prevent burnout and enhance carer efficacy, services must provide formal training and ongoing support. As shown in several reviewed interventions [32,39], providing carers with specific skills in behavioural management, communication, and navigating the health system leads to better outcomes for both the carer and the patient.
- Policymakers should consider developing comprehensive training programs and increasing financial and respite support for family carers. Healthcare organisations must allocate dedicated staff time and resources to facilitate meaningful partnerships between families and professionals, which are critical for effective collaboration.
- Future models should standardise the inclusion of family carers while allowing for flexibility in the implementation to meet the diverse and complex needs of individual OPNMHCs and their families. Adopting such a structured yet person-centred approach can significantly enhance outcomes for OPNMHCs and reduce the burden often experienced by those providing care.

The integration of family carers in the care of older patients with neurodegenerative and mental health conditions is not just beneficial but necessary for improving patient outcomes. Future models of care must prioritise family involvement, ensuring that resources, training, and support are in place to create sustainable, patient-centred care strategies.

## Figures and Tables

**Table 1:** Search concepts, keywords, MESH headings, and sample search queries for this review in CINAHL.

Step	Search Term	Results
1.	(“old n2 (‘senior citizen’ OR ‘elderly’ OR ‘older people’ OR ‘aged people’ OR ‘older patient’ OR ‘elderly patient’)”)	79,074
2.	(mh( aged+))	980,690
3.	S1 OR S2	1,005,800
4.	(‘neurodegenerative disease’ OR ‘neurodegenerative disorder’ OR ‘neurodegenerative condition’ OR ‘Alzheimer’ OR ‘dementia’ OR ‘Parkinson’ or ‘Parkinson’s disease’ or ‘Parkinsons disease’ OR ‘mental health condition’ OR ‘depression’ OR ‘anxiety’ OR ‘anxiety disorder’ or ‘major depressive disorder’ or MDD or ‘mental illness’ or ‘mental health’ or ‘mental wellbeing’ or ‘mental health disorder’ or ‘mental disorder’)	149,012
5.	S3 AND S4	181,853
6.	(‘family member’ OR ‘family-centered’ OR ‘family’ OR ‘spouse’ OR ‘brother’ OR ‘sister’ OR ‘son’ OR ‘daughter’ OR ‘relatives’ OR ‘friends’)	648,367
7.	(‘participation’ OR particip* OR ‘involvement’ OR involv* OR ‘engagement’ OR engag* OR ‘partnership’ OR ‘integration’ OR ‘support’)	1,605,722
8.	S6 AND S7	264,612
9.	S5 AND S8	1131
10.	‘primary care’ OR ‘primary health care’ OR ‘general practitioner’ OR ‘general practice’ OR ‘family medicine’ OR ‘ambulatory care’ OR ‘outpatient care’ OR ‘outpatient department’ OR ‘community care’ OR ‘community health’ OR rehabilitation clinic OR ‘home care’ OR ‘visiting nursing service’ OR ‘homebound patient’ OR ‘independent living’ OR gp OR gps OR ambulatory* OR outpatient* OR ‘in-patient’ OR ‘hospital admitted’ OR ‘intensive care unit’ OR ICU OR ‘emergency department’ OR ED OR ‘general wards’ OR ‘aged care home’ OR communit* OR ‘home nursing’ OR neighbo* OR ‘nursing service’ OR ‘allied health service’	100,824
11.	S9 AND S10	819
12.	Language: English	812
13.	Time restrictions (from 2001 onwards)	782

**Table 2:** Review inclusion and exclusion criteria.

Criteria	Inclusion	Exclusion
Population	Older patients (≥65 years) had at least one diagnosed neurodegenerative and mental health condition (i.e., Alzheimer’s, dementia, Parkinson’s disease, depression, and anxiety) Experience and perspectives of family members of community-dwelling and/or hospital-admitted OPNMHCs Healthcare professional experience and perspectives	OPNMHCs with Huntington’s disease, Lewy body diseases, spinal muscular atrophy, bipolar or psychotic disorders, or with acute, non-progressive neurological illness (e.g., stroke)
Intervention	Family-integrated health service delivery to community-dwelling and hospital-admitted OPNMHCs Family-integrated care models, management approaches, or interventions that focused on screening, treatment, or rehabilitation for OPNMHCs	Studies that did not involve family members in the interventions Interventions for family members of OPNMHCs Interventions are designed to improve management of OPNMHC behaviours and access to services
Outcomes	Experiences and views of family-integrated health service delivery, reported from the perspectives of the OPNMHCs, their family carers, and healthcare professionals.	
Study design	Quantitative, qualitative, and mixed methods studies (e.g., randomised control trials (RCTs), pre–post-test, longitudinal, quasi-experimental, cross-sectional, and case-control research designs)	Literature reviews Retrospective analysis of data Research commentary on the acceptability and feasibility of family-integrated care
Types of publication	Peer-reviewed published articles	Commentaries, book chapters, editorials, clinical guidelines, or recommendations

## Data Availability

All data are associated with this publication.

## Supplementary Materials

The following supporting information can be downloaded at: https://www.mdpi.com/article/10.3390/ijerph22071096/s1, File S1 PRSIMA checklist.
